# Supplementary material for: Active anaerobic methane oxidation and sulfur disproportionation in the deep terrestrial subsurface
Source: ISME J. 2022 Feb 16;16(6):1583–93. doi: 10.1038/s41396-022-01207-w (PMC9123182; doi:10.1038/s41396-022-01207-w)
Supplement: Supplementary file 1 — Supplementary Information [file 41396_2022_1207_MOESM1_ESM.docx]

**Supplementary Information**

**Active anaerobic methane oxidation and sulfur disproportionation in the deep terrestrial subsurface**

Emma Bell^1,5*^, Tiina Lamminmäki^2^, Johannes Alneberg^3^, Chen Qian^4^, Weili Xiong^4^, Robert L. Hettich^4^, Manon Frutschi^1^ and Rizlan Bernier-Latmani^1*^

^1^Environmental Microbiology Laboratory, Environmental Engineering Institute, School of Architecture, Civil and Environmental Engineering, École Polytechnique Fédérale de Lausanne, Lausanne, 1015, Switzerland

^2^ Posiva Oy, Eurajoki, 27160, Finland

^3^ Science for Life Laboratory, School of Engineering Sciences in Chemistry, Biotechnology and Health, Department of Gene Technology, KTH Royal Institute of Technology, Stockholm, SE-17121, Sweden

^4^ Chemical Sciences Division, Oak Ridge National Laboratory, Oak Ridge, TN 37830, United States

^5^ Present address; Department of Biological Sciences, University of Calgary, Calgary, AB T2N 1N4, Canada

*Emma Bell and Rizlan Bernier-Latmani

**Email:** [emma.bell1@ucalgary.ca](mailto:emma.bell1@ucalgary.ca) and [rizlan.bernier-latmani@epfl.ch](mailto:rizlan.bernier-latmani@epfl.ch)

### Sample site

OL-KR13 was drilled during 04/04/2001–05/05/2001. The drillhole was open until a multipacker system was installed on 07/09/2007 which is in place to date. In open drillhole conditions the flow direction is from drillhole to bedrock and the transmissivity of the fracture is ~3.3 × 10^-7^ m^2^/s (measured in 2010). The packer system has been in place for 10 years, so minimal contamination from the open drillhole phase is expected. Long-term pumping of the section sampled during this study began on 07/01/2016, two months prior to the first microbiological sample being taken on 07/03/2016. Chemical parameters (pH, dissolved O_2_, oxidation-reduction potential, conductivity) were continuously monitored to ensure that the groundwater was representative of the fracture. Chloride and sulfate values for this section (330.52–337.94) were also consistent with the baseline values (Fig. 1 in main text). Hydrological models have shown that drillhole OL-KR13 intersects a fracture zone (OL-BFZ045) that also intersects with ONKALO (Supplementary Figure 1), the underground nuclear waste repository that is currently under construction and that extends to ~455 m depth [1]. This connection could result in slow groundwater flow caused by drawdown towards open tunnels via the fracture zone.


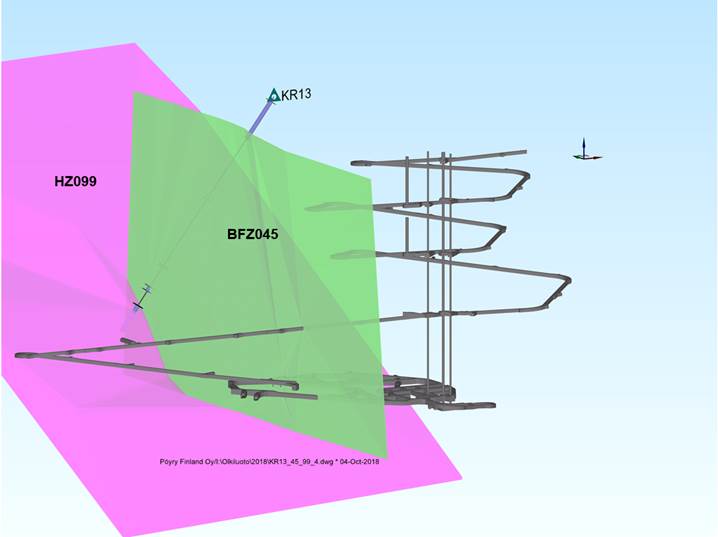


**Supplementary Figure 1:** Fracture zones intersecting drillhole OL-KR13 and ONKALO [1].

### References

1. Vaittinen T, Ahokas H, Nummela J, Paulamäki S. Hydrogeological Structure Model of the Olkiluoto Site – Update in 2010, Posiva Report 2011-65. 2011.

Available at: <https://inis.iaea.org/collection/NCLCollectionStore/_Public/43/107/43107429.pdf>
